# Evaluación de tres PCR cuantitativas para la detección de leptospiras patógenas en animales domésticos en Nicaragua

**DOI:** 10.7705/biomedica.5170

**Published:** 2020-12-10

**Authors:** Byron Flores, Nabil Halaihel, Tania Pérez-Sánchez, Jessica Sheleby-Elías, Brenda Mora, Héctor Fuertes, William Jirón

**Affiliations:** 1 Centro Veterinario de Diagnóstico e Investigación, Escuela de Ciencias Agrarias y Veterinarias, Universidad Nacional Autónoma de Nicaragua, León, Nicaragua Escuela de Ciencias Agrarias y Veterinarias Universidad Nacional Autónoma de Nicaragua León Nicaragua; 2 Departamento de Patología Animal, Facultad de Veterinaria, Universidad de Zaragoza, Zaragoza, España Universidad de Zaragoza Departamento de Patología Animal Facultad de Veterinaria Universidad de Zaragoza Zaragoza Spain

**Keywords:** Leptospira, leptospirosis/diagnóstico, reacción en cadena de la polimerasa, animales domésticos, Nicaragua, Leptospira, leptospirosis/diagnosis, polymerase chain reaction, animals, domestic, Nicaragua

## Abstract

**Introducción.:**

En Nicaragua es necesario estandarizar pruebas moleculares como la PCR en tiempo real *(quantitative Polymerase Chain Reaction,* qPCR) que mejoren el diagnóstico de leptospirosis en humanos y animales.

**Objetivo.:**

Evaluar tres qPCR para la detección de leptospiras patógenas en animales domésticos de Nicaragua.

**Materiales y métodos.:**

Se diseñaron cebadores para la amplificación del gen *LipL32* en SYBR Green (SYBR Green-A) y TaqMan, y en otros descritos previamente (SYBR Green-B). Las secuencias de 12 cepas obtenidas de la base de datos del *National Center for Biotechnology Information* (NCBI) se alinearon para la búsqueda de sondas y cebadores. La sensibilidad analítica se determinó calculando el equivalente genómico detectable, se utilizaron 18 cepas de referencia para la sensibilidad diagnóstica y 28 controles negativos para la especificidad. Los métodos se aplicaron en 129 muestras de orina de animales domésticos.

**Resultados.:**

En SYBR Green-A se obtuvo un límite de detección de cuatro equivalentes genómicos; en TaqMan, la sensibilidad fue del 94,4 % (IC_95%_ 81,1-100,0). Con SYBR Green-A, se obtuvo una sensibilidad del 77,8 % (IC_95_% 55,8-99,8), en tanto que con SYBR Green-B fue del 61,1 % (IC_95%_ 35,8-86,4). En las tres pruebas se logró una especificidad del 100 % (IC_95%_ 98,2-100,0). El 26,4 % de las muestras de animales domésticos fueron positivas con SYBR Green-A y el 6,2 % con SYBR Green-B.

**Conclusiones.:**

El SYBR Green-A presentó un límite de detección bajo, en tanto que las tres técnicas evaluadas mostraron alta especificidad, en tanto que la TaqMan tuvo la mayor sensibilidad.

## Abstract (en)

**Introduction:**

Molecular biology diagnostic methods such as real-time PCR should be used in Nicaragua to improve the diagnosis of leptospirosis in humans and animals.

**Objective:**

To evaluate three qPCR methods for pathogenic *Leptospira* detection in domestic animals.

**Materials and methods:**

Real-time PCR primers were designed for the amplification of specific regions from the *Lip 32* gene of *Leptospira* in SYBER Green (SYBER Green-A) and TaqMan, as well in SYBER Green-B as previously published. The sequences of 12 strains obtained from the database of the National Center for Biotechnology Information (NCBI) were aligned to select probes and primers. The analytical sensitivity was determined by calculating the detectable genomic equivalent while 18 pathogenic references strains and 28 negative controls were used to evaluate the sensitivity and specificity of each one of the three sets in 129 urine samples of domestic animals.

**Results:**

The detection limit of four genomic equivalents per reaction was obtained from SYBR Green-A. The specificities were 94.4% (95% CI: 81.1-100.0) for TaqMan, 77.8% (95% CI: 55.8-99.8) for SYBR Green-A, while for SYBR Green-B it was 61.1% (95% CI: 35.886.4). In the three tests, we obtained a specificity of 100% (95% CI: 98.2-100.0). In the field samples, 26.4% were positive with SYBR Green-A and 6.1% with SYBR Green-B.

**Conclusion:**

SYBR Green-A presented the lowest detection limit while the three techniques under evaluation showed high specificity while TaqMan was the most sensitive.

La leptospirosis es una enfermedad zoonótica causada por especies patógenas del género *Leptospira* que afecta principalmente a los países en desarrollo ^1^. La espiroqueta se mantiene en los portadores crónicos, que la secretan en el medio ambiente, y la infección en el humano resulta por el contacto directo con animales infectados o, indirectamente, con un ambiente contaminado ^2^^,^^3^. En Nicaragua han ocurrido varios brotes de leptospirosis humana desde 1995, los más notables en 1998 y en el 2007, y fue el segundo país en el que se observó un cuadro de hemorragia pulmonar en etapa terminal en uno de los municipios del occidente del país ^4^.

Los métodos convencionales, como el cultivo y la prueba de aglutinación microscópica *(Microscopic Agglutination Test,* MAT), no son útiles para el diagnóstico temprano, pues el cultivo de *Leptospira* spp. es difícil dado que se trata de bacterias de crecimiento lento y los anticuerpos anti*-Leptospira* solo pueden detectarse después de la segunda semana del inicio de los síntomas ^3^. Además, la prueba de aglutinación microscópica requiere el mantenimiento de leptospiras vivas y muestras de suero del convaleciente para obtener resultados concluyentes ^5^. La qPCR es más sensible que el cultivo para la detección de leptospiras en muestras clínicas. Además, se informa que su sensibilidad en estas muestras es mejor que la de la prueba de aglutinación microscópica y el cultivo ^1^^,^^3^.

En Nicaragua se han hecho esfuerzos para mejorar la respuesta frente a brotes de leptospirosis, sin embargo, continúa siendo una de las zoonosis más importantes y aún debe mejorarse el diagnóstico de laboratorio para mejorar el manejo de los casos. La qPCR puede detectar leptospiras patógenas en pacientes con resultados falsos negativos en el cultivo debido al tratamiento con antibióticos ^1^.

El propósito de este trabajo fue evaluar tres qPCR para la detección de leptospiras patógenas en animales domésticos en Nicaragua. El diagnóstico adecuado de animales portadores es esencial para establecer medidas preventivas.

## Materiales y métodos

### Extracción del ADN de las cepas de referencia

Las cepas de referencia de leptospiras se reactivaron a 30 °C en medio líquido de Ellinghausen, McCullough, Johnson y Harris (EMJH); después de siete días de incubación, se tomaron 5 ml que fueron centrifugados a 17.500*g* durante 10 minutos; se usaron 200 µl del sedimento para la extracción de ADN, siguiendo las instrucciones del fabricante (UltranClean Blood Spin MO BIO, USA).

### Diseño de cebadores

Se diseñaron cebadores para la amplificación del gen *LipL32* en SYBR Green-A y en TaqMan, así como los cebadores descritos por Levett, *et al.*^6^ (SYBR Green-B). Se utilizaron secuencias de 12 cepas obtenidas de la base de datos del *National Center for Biotechnology Information* (NCBI): 1) *Leptospira interrogans,* serovar Hardjo, cepa RTCC2821 (No. JN886739.1); 2) *L.interrogans,* serovar, Grippotyphosa, cepa RTCC2808 (No. JN886738.1); 3) *L interrogans,* serovar Hebdomadis, (No. GU220823.1); 4) *L. interrogans,* serovar Canicola, (No. DQ092412.1); 5) *L. interrogans,* serovar Lai (No. LIU89708); 6) *L.interrogans,* serovar Pomona, cepa RZ11 (No. AY461910. 1); 7) *L.noguchii,* serovar Nicaragua, cepa 1011 (No. AY461918.1); 8) *L.noguchii,* cepa Fort Bragg (No. AF181556); 9) *L kirschneri,* cepa 5621 (No AY461917.1); 10) *L kirschneri* (No. AF121192.1); 11) *L. kirschneri,* serovar Grippotyphosa, cepa RM52 (No. AY461915.1), y 12- *L. borgpetersenii,* serovar Hardjo-bovis (No. AM937000.1).

Estas secuencias se alinearon utilizando el algoritmo Clustal W, versión 1.6, con el programa *Molecular Evolutionary Genetics Program Analysis,* versión 6 (MEGA6) ^7^. La alineación se utilizó para encontrar una secuencia de consenso y emplearla como plantilla para el diseño de sondas y cebadores utilizando el PrimerQuest (Integrated DNA Technologies) ^8^. Posteriormente, se compararon manualmente para garantizar la ausencia de desajustes. Para buscar posibles reacciones cruzadas, los cebadores y la sonda se evaluaron con la herramienta BLAST ^9^ (cuadro 1).

**Cuadro 1:** Secuencias de los cebadores y la sonda para la detección de leptospiras patógenas

Nombre	Secuencias	Producto	PCR	Referencias
Taq32F	5´-GACAAACGAAACCGTAAA-3´			
Taq32R	5´- GTTTCCATCGACTAAACC-3			
Pr32	5´-FAM-CTGTGATCAACTATTACGGATACG-TAMRA-3´	103 bp	TaqMan	Este estudio
LipL32F	5´-GCCTAAAAAGCTCTTTTGTTC-3´	62 bp	SYBR Green-A	Este estudio
LipL32R	5´-GGACAAACGAAACCGTAAAA-3'			
LipL32-270F	5'-CGCTGAAATGGGAGTTCG TATGATT-3'			
LipL32-692R	5'-CCAACAGATGCAACGAAAG ATCCTTT-3'	423 bp	SYBR Green-B	^6^

### Mezclas y protocolos de amplificación

Para el SYBR Green-A, se utilizó un volumen de 20 µl de reacción colocando 10 µl de SYBR Green GoTaq qPCR Master Mix 2x™ (Promega, USA), 1 µl de cada cebador a 10.000 nmol/L (500 nmol/L en la reacción), 7 µl de agua libre de nucleasas y 1 µl de ADN de la muestra.

El protocolo de amplificación fue de 7 minutos a 95 °C, seguido de 44 ciclos (15 segundos a 55 °C, 15 segundos a 72 °C, 5 segundos a 79 °C y 15 segundos a 94 °C); para la curva de fusión (Tm), fue de 10 segundos a 94 °C, 5 segundos a 65 °C, seguido de un aumento y lectura de 0,5 °C hasta alcanzar 95 °C.

La reacción TaqMan se hizo en un volumen de 20 µl de reacción colocando 10 µl de sonda GoTaq qPCR Master Mix 2x™ (Promega, USA), 1 µl de cada cebador a 10.000 nmol/l (500 nmol/l en la reacción) y 1 µl de sonda a 5.000 nmol/l (2.500 nmol/l en la reacción), 6 µl de agua libre de nucleasas y 1 µl de ADN de la muestra.

El protocolo de amplificación fue de 4 minutos a 95 °C, seguido de 44 ciclos (30 segundos a 55 °C y 5 segundos a 94 °C) y un último a 4 °C. Para el SYBR Green-B, se utilizó el protocolo descrito por Levett, *et al.*^6^. Las amplificaciones se llevaron a cabo en el sistema en tiempo real CFX Connect™ (Bio-Rad, USA).

### Sensibilidad analítica

La cantidad de ADN genómico de *Leptospira* detectable se estimó midiendo la absorbancia de ADN con un espectrofotómetro BioDrop™ (Cambridge, United Kingdom) a partir de un cultivo puro de *L. interrogans,* serovar Pomona, cepa Pomona. La muestra se diluyó hasta los 20 ng/µl; se consideró que el genoma de la bacteria es, aproximadamente, de 4,6 Mb (5,1 x 10^-6^ ng) para calcular el número de unidades genómicas ^10^. De este modo, se obtuvo 1 µl de muestra que contenía 3,9 x 10^6^ equivalentes genómicos, el cual se aplicó en la primera reacción a partir de la cual se hicieron diluciones en serie de base 10 en agua libre de nucleasas, repitiendo cada dilución ocho veces en la misma placa.

Con el promedio del ciclo umbral *(cycle threshold,* Ct), se hizo una curva de regresión para determinar el número mínimo de equivalentes genómicos que la técnica fuera capaz de detectar.

### Sensibilidad diagnóstica

Se utilizaron 18 cepas de referencia de seis especies patógenas como controles positivos, distribuidas así: siete cepas de *L. interrogans,* dos de *L. borgpetersenii,* tres de *L. noguchii,* tres de *L. weilii,* dos de *L. kischneri* y una de *L. santarosai.*

### Especificidad

Para detectar reacciones falsas positivas, se emplearon 28 controles negativos, incluidas cuatro cepas de *Staphylococcus aureus,* dos de *Escherichia coli,* cuatro de *Salmonella* spp., cuatro de *Streptococcus pyogenes,* y dos de *Proteus* spp. También, se emplearon muestras de ADN positivas para otros agentes, entre ellas, dos muestras positivas para *Brucella* spp., dos para *Rickettsia* spp., dos para *Borrelia burgdorferi,* y dos para *Bartonella henselae.* También, se utilizaron las cepas de *L. biflexa* (Patoc1) y *L. meyeri* (Veldrat) como especies saprofitas.

### Análisis de muestras de animales domésticos

Se tomaron muestras de orina de animales que cumplieran con los siguientes criterios: a) animales de especies domésticas; b) que se encontraran cerca (radio de 100 m) de casos de leptospirosis humana confirmados por el Ministerio de Salud de Nicaragua durante brotes ^11^; c) sin sintomatología de leptospirosis; d) con más de tres meses de permanencia en el lugar antes del muestreo, y e) que los propietarios dieran el consentimiento informado.

Se analizaron 129 muestras que incluyeron 49 animales bovinos, 45 perros, 26 cerdos, 7 caballos y 2 ovejas. Para la extracción de ADN, se tomaron 5 ml de orina que fueron centrifugados a 1.500g durante 10 minutos, y se usaron 200 µl del sedimento para la extracción de ADN, siguiendo las instrucciones del fabricante (UltranClean Blood Spin MO BIO, USA). Estas muestras fueron analizadas con SYBR Green-A y SYBR Green-B.

### Análisis estadísticos

La sensibilidad, la especificidad y el intervalo de confianza del 95 % (IC_95%_) se obtuvieron utilizando el método exacto de Fisher con el programa Epidat 3.1 ^12^; el índice Kappa se utilizó para comparar los resultados de las qPCR, la prueba de Pearson para las correlaciones entre los ciclos umbral y, en el límite de detección, se empleó una regresión lineal.

## Resultados

Con la TaqMan se amplificaron 17 de las 18 cepas de referencia de las especies patógenas para obtener una sensibilidad del 94,4 % (IC_95%_ 81,1100,0), en SYBR Green-A; 14 de 18 cepas se amplificaron y se obtuvo una sensibilidad de 77,8 % (IC_95%_ 55,8-99,8), en tanto que, en el SYBR Green-B, 11 de 18 cepas fueron positivas, para una sensibilidad de 61,1 % (IC_95%_ 35,886,4). En las tres pruebas, la especificidad fue de 100 % (IC_95%_ 98,2-100,0), ya que no se observó amplificación en los 28 controles negativos, incluidas las cepas Patoc1 y Veldrat (no patógenas) (cuadro 2).

**Cuadro 2:** Resultados de las tres PCR en las 20 cepas de referencia

Especie	Serovares	Cepas	TaqMan	SYBR Green LipL32-A	SYBR GreenLipL32-B
*L. interrogans*	Australis	Ballico	Positivo	Positivo	Positivo
*L. interrogans*	Canicola	Hond Utrecht IV	Positivo	Positivo	Positivo
*L. interrogans*	Djasiman	Djasiman	Positivo	Positivo	Positivo
*L. interrogans*	Icterohaemorrhagia	RGA	Positivo	Positivo	Positivo
*L. interrogans*	Icterohaemorrhagia	Kantorowic	Positivo	Positivo	Positivo
*L. interrogans*	Pomona	Pomona	Positivo	Positivo	Positivo
*L. interrogans*	Hardjo	Hardjoprajitno	Positivo	Negativo	Negativo
*L. noguchii*	Nicaragua	1011	Positivo	Positivo	Negativo
*L. noguchii*	Louisiana	LSU 1945	Positivo	Positivo	Positivo
*L. noguchii*	Panama	CZ 214	Positivo	Positivo	Positivo
*L. borgpetersenii*	Javanica	Veldrat Batavia	Positivo	Positivo	Positivo
*L. borgpetersenii*	Mini	Sari	Positivo	Negativo	Negativo
*L. weilii*	Sarm111	Sarmin	Positivo	Positivo	Positivo
*L. weilii*	Celledoni	Celledoni	Negativo	Positivo	Negativo
*L. weilii*	Qingshui	L 105	Positivo	Positivo	Positivo
*L. kirschneri*	Cynopteri	3522 C	Positivo	Negativo	Negativo
*L. kirschneri*	Grippotyphosa	Moskva V	Positivo	Negativo	Negativo
*L. meyeri*	Semaranga	Veldrat^*^	Negativo	Negativo	Negativo
*L. santarosai*	Shermani	1342 K	Positivo	Positivo	Negativo
*L. bifhxa*	Patoc	Patoc 1^*^	Negativo	Negativo	Negativo

^*^ Cepa no patógena

Entre la TaqMan y el SYBR Green-A se observó un kappa de 0,305 (bajo), entre la TaqMan y el SYBR Green-B fue de 0,355 (bajo), en tanto que, entre el SYBR Green-A y el SYBR Green-B, el valor fue de 0,668 (bueno) (cuadro 3).

**Cuadro 3:** Concordancia entre las tres qPCR

qPCR		TaqMan		SYBR Green-A		SYBR Green-B	
	Resultados	Negativo	Positivo	Negativo	Positivo	Negativo	Positivo
TaqMan	Negativo	-	-	2	1	3	0
	Positivo	-	-	4	13	6	11
SYBR Green-A	Negativo	0,306 (0,202)		-	-	6	3
	Positivo			-	-	0	11
SYBR Green-B	Negativo	0,355 (0,038)*		0,668 (0,001)^**^		-	-
	Positivo					-	-

En la parte superior derecha se presentan los números absolutos y en la inferior, los resultados de las pruebas estadísticas. Fuera del paréntesis se muestran los valores de kappa y, dentro, los valores de p.

* Significativo a 0,05 (IC_95%_)

* ^*^ Significativo a 0,01 (IC_99%_)

Se encontró una correlación positiva muy marcada en los ciclos umbral observados en la TaqMan y el SYBR Green-A (regresión lineal, R^2^=0,937; p<0,01).

**Figura 1:**
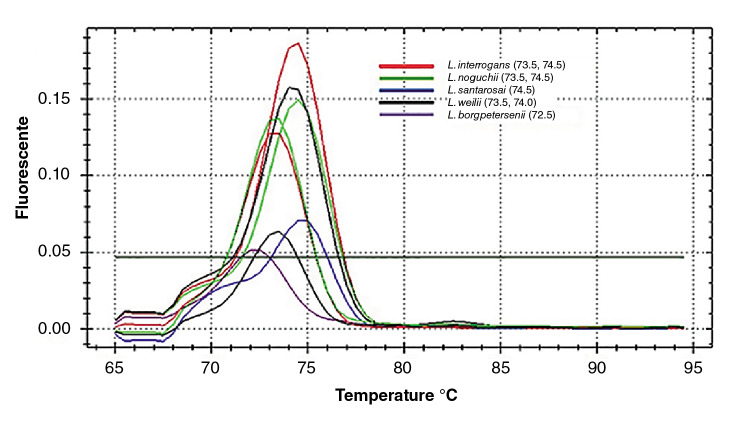
Temperaturas de fusión (Tm) por especie de leptospiras patógenas en el SYBR Green-A. Se muestran las curvas de fusión para cinco especies de leptospiras patógenas, en las cuales no se encontró asociación entre temperatura y especie de *Leptospira,* de tal manera que las especies de *L. interrogans* (rojo) proporcionaron diferentes Tm, al igual que las observadas en *L. noguchii* (verde).

En el análisis de las curvas de fusión (Tm) de los productos amplificados con SYBR Green-A, se encontraron valores entre los 72,5 y 74,5 °C. El análisis de las Tm de las seis cepas de *L. interrogans* reveló que tres de ellas presentaron una temperatura de 73,0 °C, dos de 73,5 °C y una de 74,5 °C. Las tres cepas de la especie *L. noguchii* mostraron Tm diferentes, lo que es similar a lo observado en las tres cepas de la especie *L. weilii.* No se encontró asociación entre las Tm y la especie de *Leptopira* (p>0,05) (figura 1).

**Figura 2:**
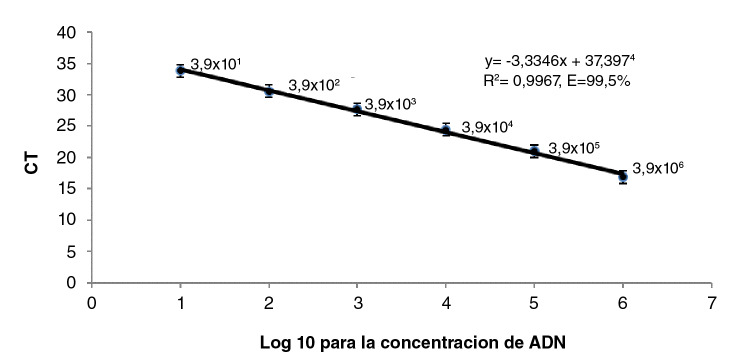
Curva estándar para los ciclos umbral y la concentración de ADN en SYBR Green-A. Se muestran los valores de ciclos umbral obtenidos en seis diluciones en serie de la base 10 a partir de una concentración conocida de ADN (3,9 x 106 equivalentes genómicos por µl) obtenida de un cultivo puro de cepa Pomona. Se observó un aumento de 3,33 en los ciclos umbral para cada dilución.

Con el SYBR Green-A, se obtuvo un límite de detección de 4 equivalentes genómicos por reacción, una eficiencia de 99,55 % y una R^2^ de 0,99 (alta correlación) (figura 2). En las muestras de campo, los resultados revelaron que, con la técnica SYBR Green-B, fueron positivas 8 de las 129 (6,2 %) muestras, en tanto que, con SYBR Green-A, la frecuencia de positivos fue de 34 en 129 (26,4 %).

## Discusión

La sensibilidad diagnóstica fue mayor con la TaqMan (94,4 %), y la especificidad fue alta con las tres técnicas. El valor correspondiente a la mayor sensibilidad de la TaqMan difiere de lo descrito por Cao, et *al.,* en su estudio ^13^, quienes señalan que el uso de sondas arrojó una mayor especificidad que el SYBR Green, pero una menor sensibilidad, ya que se ven más fácilmente afectadas por factores inhibitorios y requieren la complementariedad perfecta, no solo en los dos cebadores, sino también en la sonda.

El fluoróforo SYBR Green puede dar resultados cuantitativos erróneos si no se eliminan productos inespecíficos o dímeros de cebadores ^13^; sin embargo, es una técnica simple y económica en comparación con la TaqMan. Aunque a veces el análisis de la Tmcon SYBR Green se considera menos específico en comparación con el uso de sondas, esta técnica puede proporcionar una especificidad similar si los cebadores están diseñados apropiadamente y se hace una estandarización adecuada ^14^.

Los resultados heterogéneos entre las tres qPCR en el gen *LipL32* de una misma especie de leptospira podrían explicarse con lo planteado por Haake, *et al.*^15^, en su estudio de las secuencias de nucleótidos del gen *LipL32,* en el que la mayoría de los polimorfismos detectados en las secuencias fue silenciosa (50 de 73), lo que sugiere que existe una presión evolutiva para mantener la configuración primaria de la proteína LipL32, pero no las secuencias de nucleótidos, y ello puede dificultar la detección de todas las cepas con un solo par de cebadores.

En este estudio, se obtuvo una mejor sensibilidad diagnóstica con ampliaciones pequeñas en la TaqMan (103 pb) y el SYBR Green-A (62 pb) que en el SYBR Green-B (423 pb), lo que podría estar relacionado con la disminución de la eficiencia de la qPCR en ampliaciones mayores de 150 pb debido a que se requiere más tiempo para completar el producto ^16^.

Se obtuvo una correlación positiva muy marcada entre la TaqMan y el SYBR Green-A en los Ct obtenidos; sin embargo, se observaron valores de ciclos umbral más altos con el SYBR Green-A, lo que podría estar asociado con la mayor sensibilidad para el diagnóstico mostrada por la TaqMan. En el estudio de Cao, *et al.*^13^, se registraron valores de ciclos umbral más elevados con la TaqMan que con el SYBR Green al amplificar el gen *LipL32.*

El análisis de las Tm obtenidas con el SYBR Green-A no evidenció asociación con la especie. Los resultados del análisis de las Tm en la qPCR descritos por Levett, *et al.*^6^, fueron similares a los del presente estudio, ya que obtuvieron la misma Tm (84 °C) para las especies *L. interrogans, L. kirschneri* y *L. noguchii.* Por otro lado, en el estudio de Merien, *et al.*^14^, el análisis de las Tm en la amplificación del *locus* lfb1 evidenció la capacidad para diferenciar entre las especies de leptospiras, lo que podría deberse a la heterogeneidad dentro de la secuencia del *locus* lfb1 y a la amplificación de un segmento más grande, lo que permite una mayor probabilidad de combinaciones en el porcentaje de G-C entre las especies.

El SYBR Green-A presentó un límite de detección de 4 equivalentes genómicos por reacción, similar a lo descrito por Levett, *et al.*^6^, quienes encontraron una sensibilidad analítica de 3 equivalentes genómicos por reacción en muestras de sangre y de 10 equivalentes genómicos por reacción en muestras de orina. La eficiencia y la regresión lineal (R^2^) fueron altas (figura 2), lo que podría atribuirse a una buena técnica de extracción de ADN con bajas concentraciones de sal y otros factores inhibidores en el medio EMJH, así como al tamaño apropiado del amplicón, ya que, como lo describen Opel, *et al.*^17^, estos pueden ser factores que alteran la eficiencia de la técnica y, por lo tanto, su capacidad para cuantificar adecuadamente el número de copias de ADN.

En las muestras de campo, el porcentaje de muestras positivas con SYBR Green-A fue similar al descrito por otros autores en muestras de orina de animales en granjas de Brasil ^18^. Por otro lado, un cultivo previo de las muestras en EMJH, puede aumentar la sensibilidad, pero tiene la desventaja de requerir más tiempo.

Cada vez más, el diagnóstico de leptospirosis se basa en la qPCR, que proporciona un diagnóstico preciso antes de que aparezca la seroconversión tanto en animales como humanos, con las consecuentes alteraciones en el sistema inmunitario ^19^. Sin embargo, Picardeau *et al.*^3^, señalan que una qPCR positiva demuestra la presencia de leptospiras patógenas, aunque apenas predice el serovar. También hay estudios, como el de Agampodi, *et al.,* en que se ha demostrado que la carga bacteriana detectada por qPCR no está relacionada con la gravedad del cuadro clínico y, además, que debe determinarse el momento más apropiado para tomar las muestras, con el fin de obtener una mejor sensibilidad ^5^.

Las qPCR diseñadas y aplicadas a muestras de animales de la región facilitarán una vigilancia más efectiva y oportuna para desarrollar las medidas apropiadas de prevención de la leptospirosis. Las técnicas evaluadas en este estudio representan una alternativa para el diagnóstico temprano de animales portadores y contribuyen al sistema de salud del país.
